# The Combined Toxic and Genotoxic Effects of Cd and As to Plant Bioindicator *Trifolium repens* L

**DOI:** 10.1371/journal.pone.0099239

**Published:** 2014-06-10

**Authors:** Alessandra Ghiani, Pietro Fumagalli, Tho Nguyen Van, Rodolfo Gentili, Sandra Citterio

**Affiliations:** Department of Earth and Environmental Sciences, University of Milano-Bicocca, Milan, Italy; University of Milano Bicocca, Italy

## Abstract

This study was undertaken to investigate combined toxic and genotoxic effects of cadmium (Cd) and arsenic (As) on white clover, a pollutant sensitive plant frequently used as environmental bioindicator. Plants were exposed to soil spiked with increasing concentrations of cadmium sulfate (20, 40 and 60 mg Kg^−1^) or sodium arsenite (5, 10 and 20 mg Kg^−1^) as well as with their combinations. Metal(loid) bioavailability was assessed after soil contamination, whereas plant growth, metal(loid) concentration in plant organs and DNA damage were measured at the end of plant exposition. Results showed that individual and joint toxicity and genotoxicity were related to the concentration of Cd and As measured in plant organs, and that As concentration was the most relevant variable. Joint effects on plant growth were additive or synergistic, whereas joint genotoxic effects were additive or antagonistic. The interaction between Cd and As occurred at both soil and plant level. In soil the presence of As limited the bioavailability of Cd, whereas the presence of Cd increased the bioavailability of As. Nevertheless only As biovailability determined the amount of As absorbed by plants. The amount of Cd absorbed by plant was not linearly correlated with the fraction of bioavailable Cd in soil suggesting the involvement of additional factors, such as plant uptake mechanisms. These results reveal that the simultaneous presence in soil of Cd and As, although producing an additive or synergistic toxic effect on *Trifolium repens* L. growth, generates a lower DNA damage.

## Introduction

The use of efficient early warning bioindication systems represents a powerful approach for assessing and interpreting the impact of natural or anthropogenic perturbations in soil ecosystems preventing environmental alteration and human disease.

Living organisms provide information on the cumulative effects of environmental stressors and as such bioindication is complementary to direct physical and chemical measurements [1]. *T. repens* is a pollutant-sensitive plant, suitable for biomonitoring campaigns. Specifically, its environmental exposition followed by a DNA analysis with molecular markers allows the detection of sub-lethal levels of genotoxic compounds in the environment [2]. However, given the limited information available on the joint-genotoxic-effect of chemicals, the interpretation of biomonitoring results is often difficult. In addition, most environmental risk assessments of contaminated lands are currently based on guideline values derived from the ecotoxicological properties of specific chemicals, whereas it is well known that environmental pollutants interact producing additive, antagonistic or synergistic effects on exposed organisms [3]–[7]; it is then evident that there is a clear need to improve the knowledge about the combined effects of stressors on bioindicators.

In this work we considered the combined toxic and genotoxic effects of Cd and As, two of the most dangerous pollutants for both environment and human health. They are ranked among the top ten priority hazardous compounds by the Agency for Toxic Substances and Disease Registry [8]. Over the last century, their natural presence on the earth's crust along with improper industrial and agricultural practices has led to the pollution of many areas, which are now of public concern. In fact, since Cd and As cannot be degraded by living organism, after their natural or anthropogenic release, they persist in soil and sediments where they can accumulate up to harmful levels. As consequence, detrimental effects on life in the environment and on human health are exerted by these two elements directly or indirectly, through the food chain [9].

Among the damages induced by As and Cd to living organisms, genome alteration is one of the most dangerous. Genomic instability is particularly related to cancer induction and progression in animals and to inhibition of growth and even to death in plants. In wild and agro ecosystems the presence of genotoxic compounds significantly reduces the number of species and decreases the yield and quality of crops [4], [10].

Many in vitro and in vivo studies demonstrated Cd and As induction of micronuclei, chromosomal aberrations, DNA strand breaks and oxidative DNA base damage [11].

Moreover the two elements are classified as Group 1 carcinogens by the International Agency for Research on Cancer (IARC). The human exposure to Cd or As has been associated with various cancers, principally kidney and lung for Cd [12] and skin, lung, liver and bladder for As [11]. The underlying cellular pathways leading to cancer are similar for the two metals and include mechanisms inducing DNA damage, such as oxidative stress and DNA repair inhibition. Thus the available literature clear shows that Cd and As are genotoxic compounds and that they exert their effect even at low concentrations. However most of this information comes from studies on single chemical whereas no data are available on their genotoxic joint action. In our experiment the bioindication system, based on the use of *T. repens*, as bioindicator, and molecular markers, as suitable tool to detect DNA changes, were used to investigate the combined toxic and genotoxic effect of increasing concentrations of Cd and As in soil. Effects were also correlated to the uptake and distribution of Cd and As in plant organs.

## Materials and Methods

### Plant bioindicator and experimental design

*T. repens* seeds cv. Ladino (Ingegnoli, Milan, Italy) were surface sterilized and directly grown in soil containing 3.0% organic matter for 30 days.

For the exposition to Cd or As, the nearly 5 cm high plantlets obtained after 30 days from germination, were transferred to pots filled with 2.0 kg of soil contaminated with or without (control) increasing concentrations of cadmium sulfate (20, 40 and 60 mg Kg^−1^ Sigma, St. Louis, MO) or sodium arsenite (5, 10 and 20 mg Kg^−1^; Sigma). Concentrations were defined on the basis of the results obtained by preliminary trials by which the sensitivity of *T. repens* plantlets to cadmium sulfate and sodium arsenite was tested.

For the joint-exposition to Cd and As, the following 9 soils were prepared by combining the 3 concentrations of cadmium sulfate and sodium arsenite, used to prepare soils for the single-expositions: (1) Cd 20 and As 5 mg Kg^−1^, (2) Cd 20 and As 10 mg Kg^−1^, (3) Cd 20 and As 20 mg Kg^−1^, (4) Cd 40 and As 5 mg Kg^−1^, (5) Cd 40 and As 10 mg Kg^−1^, (6) Cd 40 and As 20 mg Kg^−1^, (7) Cd 60 and As 5 mg Kg^−1^, (8) Cd 60 and As 10 mg Kg^−1^, (9) Cd 60 and As 20 mg Kg^−1^.

For each treatment, three different pots with 15 plantlets were prepared and placed in a growth room for 2 weeks (25°C; 10 h dark/14 h light, 150 µmol m^−2^ s^−1^ ).

At the end of exposition, plant survival and growth were determined and RAPD analysis was carried out to evaluate DNA damage. Three independent repetitions of the entire experiment were performed.

### Plant survival and growth measurements

The assessment of plant survival and plant growth was performed after exposition. Plant growth was evaluated by measuring plant organ dry weight (DW): for each treatment the shoots and roots from 30 plants were placed in a drying cabinet at 40 °C until a constant weight was reached. Statistical analyses were performed using the GraphPad Prism software for Windows (version 4.0 GraphPad Software Inc., San Diego CA): ANOVA and Dunnet or Tukey test were applied to the data when normality and homogeneity of variance were satisfied. Data which did not conform to the assumptions were alternatively transformed into logarithms, or were analysed by Kruskal Wallis non-parametric procedures.

### Analytical methods for metal(loid) quantification

Before plant exposition, the bioavailable fraction of Cd and As was quantify in control and contaminated soils following the protocol of Lindsay and Norwell [13]. Briefly, 5 g of soil were extracted with 10 ml of 5 mM DTPA (Sigma-Aldrich), 0.1 M trietanolamine (Sigma-Aldrich) and 0.01 M CaCl_2_ (Sigma-Aldrich), for 2 h at 20°C under stirring. Samples were then filtered and metal concentrations were determined by graphite furnace atomic absorption spectroscopy (GFAAS; AAnalyst600, Perkin-Elmer). Nine soil samples for each treatment were processed.

Cd and As were also quantified in plant organs applying the USEPA 3051a protocol. The harvested plants were carefully washed with tap water and then with distilled water to remove soil debries before analysis. All the samples were dried at 100 °C overnight. For each sample 10 mL of HNO3 and 2 mL of HClO3 were added to 0.2 g of dry plant matter. The samples were digested by using the ETHOS HPR 100/10 microwave lab station (FKV, Bergamo, Italy) reaching the 180°C temperature. After their complete mineralization, they were opportunely diluted and analysed by graphite furnace atomic absorption spectroscopy (GFAAS; AAnalyst600, Perkin-Elmer). Standards (from ENEA Research Centre, Roma, Italy) and blanks were run with all sample series for quality control.

### Random amplified polymorphic DNA (RAPD)

RAPD technique was used to quantify DNA sequence changes in test-plants. DNA was isolated separately from shoots and roots by using DNeasy isolation and purification kit (Qiagen, Italy). The kit was used to obtain high quality DNA, free of polysaccharides or other metabolites which might interfere with DNA amplification. At least three independent extractions were performed for each treatment.

Extracted genomic DNA and twelve different 10-bp-long random primers were used in RAPD-Polymerase chain reaction (RAPD-PCR); the sequences of the primers are reported in Table S1. DNA amplification was performed in a 20 µl final reaction volume containing 15 ng genomic DNA, 1 µM primer and 1X Taq PCR Master Mix (Qiagen). The RAPD amplification protocol consisted of an initial denaturing step of 5 min at 95°C, followed by 45 cycles at 95°C for 30 sec (denaturation), 35°C for 30 sec (annealing) and 72°C for 30 sec (extension), with an additional extension period of 8 min at 72°C. DNA amplification products were separated on a ethidium bromide-stained 2.0% high resolution agarose gel (Sigma-Aldrich), using a Tris-borate-EDTA (TBE) buffer (90 mM Tris base, 90 mM boric acid and 2 mM EDTA). Three independent replicates were performed for each sample.

Visual inspection under UV light of the resulting gels, allowed for scoring of polymorphic bands. For statistical analysis, each RAPD band detected after electrophoresis of the amplification DNA products was scored as a binary character for its absence (0) or presence (1). The percentage of polymorphism (P%), that represents the ratio between the number of polymorphic bands and total detected bands X 100, was determined for each sample and data were statistically analysed using the software program GraphPad Prism for Windows version 4.0 (GraphPad Software Inc., San Diego CA USA). The statistical significance between the treated samples and the control were obtained by applying ANOVA and Dunnet test (P<0,05).

### Statistical determination of Cd and As interaction type

The interaction type existing between Cd and As in each treatment and concerning their joint effect on plant growth and DNA sequence change were evaluated by applying the statistical method reported by Ince et al. [14]. The method was based on testing the null hypothesis of ‘‘additive effect’’ at 95% confidence level.

Specifically, the interaction of Cd and As in each treatment was assessed by comparing the observed toxicity at the i^th^ test level and at the concentration (x+y)i (where x and y were the concentrations of the first and second element, respectively) with the value of the null hypothesis at that level, defined as ‘‘the sum of the toxicity indices of the two elements, tested previously at x and y’’.

For the joint effect on plant growth, evaluation of the null hypothesis was based on multiplication of plant dry weigh (PDW) of each element as percentage of control, whereas for the joint effect on DNA sequence changes the null hypothesis was evaluated by the addition of plant damage induced by each element, defined as PP  =  polymorphism percentage. Thus, toxic and genotoxic interactions at each binary test level were assessed by statistical testing of the two null hypotheses PDW_H_ and PP_H_, defined by Equation 1 and Equation 2 for growth and DNA damage data, respectively:
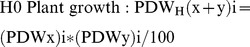
(1)

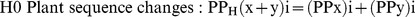
(2)where (x+y)_i_ was the i^th^ combination of Cd and As concentrations in soil, (PDWx)i and (PDWy)i the plant dry weight (as%) for each metal ion, recorded at the xi^th^ and yi^th^ singular concentrations, and (PPx)i, (PPy)i the percentage of polymorphism induced by each element, recorded at the xi^th^ and yi^th^ singular concentrations.

The compound interactions were called ‘‘antagonistic,’’ ‘‘additive,’’ or ‘‘synergistic’’ according to the statistical significance (t student) and the sign of the difference between the tested hypothesis and the value of the observed effect.

Regression and Redundance statistical analyses (RDA) were also applied to investigate the relationships between variables and their relevance to the joint-effects of Cd and As.

## Results

### Bioavailability of Cd and As in soil

The bioavailable amount of Cd and As in artificially contaminated soils was assessed just before *T. repens* exposition. The measured concentrations of DTPA-extractable Cd and As are reported in Table 1. In keeping with literature [15]–[18] Cd was much more bioavailable than As: the percentage of bioavailable As and Cd ranged from 0.016 to 0.055 and from 0.43 to 0.79, respectively. In soils contaminated with single compounds the bioavailable amounts of both Cd and As increased in parallel with the increase of metal concentration added to soil (r^2^_Cd_ =  0.99 r^2^_As_ =  0.97). A different bioavailability trend was instead observed in soil simultaneously contaminated with the two elements: the presence of As reduced the amounts of bioavailable Cd, whereas the presence of Cd increased the amounts of bioavailable As.

**Table 1:** Bioavailable content (µg g^−1^) of Cd and As in soil before plant exposition.

Soil Sample	pH	Bioavailabe As (µg g-1)	Bioavailabe Cd (µg g-1)
**CTR**	7.9	BDL	BDL
**As 5**	7.8	0.08±0.01	BDL
**As 10**	8	0.25±0.04	BDL
**As 20**	7.8	0.80±0.06	BDL
**Cd 20**	7.8	BDL	15.76±2.72
**Cd 40**	7.8	BDL	26.81±4.32
**Cd 60**	7.9	BDL	36.79±5.91
**As 5+Cd 20**	8	0.13±0.02	9.87±1.59
**As 5+Cd 40**	8	0.12±0.03	18.65±2.96
**As 5+Cd 60**	7.8	0.14±0.02	32.57±5.41
**As 10+Cd 20**	7.9	0.33±0.03	8.91±1.49
**As 10+Cd 40**	7.9	0.32±0.04	17.41±2.72
**As 10+Cd 60**	7.9	0.37±0.03	31.99±5.18
**As 20+Cd 20**	7.9	1.11±0.05	9.58±1.55
**As 20+Cd 40**	7.9	1.04±0.04	19.83±3.20
**As 20+Cd 60**	7.9	0.93±0.05	30.70±4.95

Data are the mean+SD of 9 soil samples (3 from each pot).

BDL: below instrument detection limit.

### Effect of Cd and As contaminated soils on plant survival and growth

**Figure 1:**
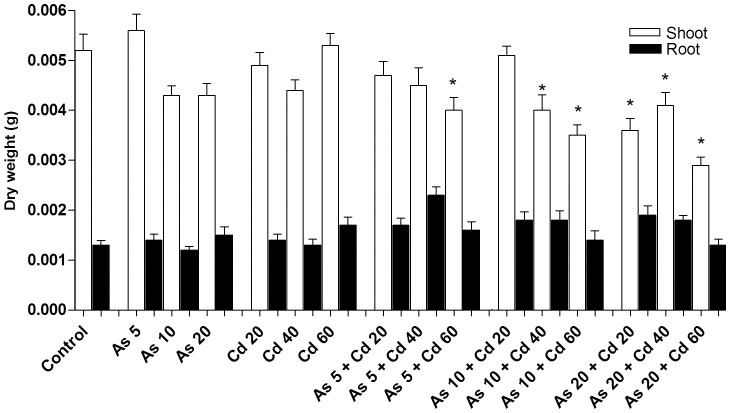
Effect of metal(loid) stress on *T. repens* growth, measured as dry weight (DW). Data are the mean of 30 measurements from single plants per each treatment. The asterisk (*) indicates statistically significant differences with respect to the control (ANOVA and Dunnet test; P<0.05).

Single and joint effects of Cd and As on plant survival and plant development were assessed after 15 days of exposition. Plant development was evaluated by measuring plant organ dry weight (DW). As expected on the basis of preliminary trials, none of the used single Cd or As concentrations negatively affected plant survival and plant DW (Fig.1). Plant survival was not affected also by all the combined treatments. On the contrary, the combination of As5 with the higher Cd concentration (Cd 60) and the combination of As10 with Cd 40 or Cd 60 and of As 20 with all the tested Cd concentrations statistically reduced the shoot development (Fig.1). Concerning the effect of these combined concentrations on roots, although a growth reduction trend was observed, the results obtained were not statistically significant, given the root very low DW and the consequent difficulty in assessment (Fig.1).

A statistiscal analysis, according to Ince et al. [14], was applied to evaluate the type of interaction existing between As and Cd, responsible for the joint effect on plant growth observed in each treatment. Table 2 shows the results of the analysis. A synergistic effect leading to plant growth reduction was found when the higher tested Cd concentration (Cd 60) was combined with As 5 or As 10 or As 20. An additive effect was instead determined for all the other soil binary mixture.

**Table 2:** Observed and calculated toxic effects at binary test combinations x:y, and single metal concentrations x, y, respectively (predicted interaction types).

	x(µg g^−1^)	y(µg g^−1^)	Observed toxicity PDW_obs_	Calculated toxicityPDW_calc_(PDW_x_ * PDW_y_/100)	Difference(PDW_obs_ - PDW_calc_)	Difference standarderror	t Student(df = 34)	Signifi cance(P<0.05)	InteractiveEffect
**SHOOT**	As	Cd							
	5	20	84.3±5.1	101.3±8.1	−17.0	9.6	−1.8	I	additive
	5	40	80.0±6.2	91.6±5.9	−11.6	8.5	−1.4	I	additive
	5	60	70.1±4.7	110.7±7.8	−40.6	9.1	−4.5	S	synergistic
	10	20	90.6±7.6	77.1±6.2	13.5	9.8	1.4	I	additive
	10	40	71.4±5.4	69.7±4.5	1.7	7.0	0.2	I	additive
	10	60	69.0±3.9	84.2±5.9	−15.2	7.1	−2.1	S	synergistic
	20	20	64.7±4.1	76.9±6.2	−12.2	7.4	−1.7	I	additive
	20	40	73.2±4.8	69.5±4.5	3.7	6.5	0.6	I	additive
	20	60	51.6±3.0	84.0±5.9	−32.4	6.6	−4.9	S	synergistic
**ROOT**	5	20	92.9±7.7	120.8±13.5	−27.9	15.5	−1.8	I	additive
	5	40	123.5±8.7	96.8±11.1	−6.8	14.1	−0.5	I	additive
	5	60	83.6±8.4	142.8±26.6	−59.2	27.9	−2.1	S	synergistic
	10	20	95.2±8.4	81.0±9.0	14.2	13.1	1.1	I	additive
	10	40	98.3±10.4	64.9±7.4	9.1	12.8	0.7	I	additive
	10	60	73.2±9.7	95.8±17.9	−.8	20.3	−2.0	I	synergistic
	20	20	103.6±10.1	97.8±10.9	5.8	14.8	0.4	I	additive
	20	40	97.6±5.6	78.4±9.0	19.3	10.6	1.8	I	additive
	20	60	69.1±6.0	115.6±21.6	−44.2	22.4	−2.0	S	synergistic

PDW: plant dry weight; S: statistically significant; I: statistically insignificant; df: degrees of freedom.

### Accumulation of Cd and As in plant organs

**Figure 2:**
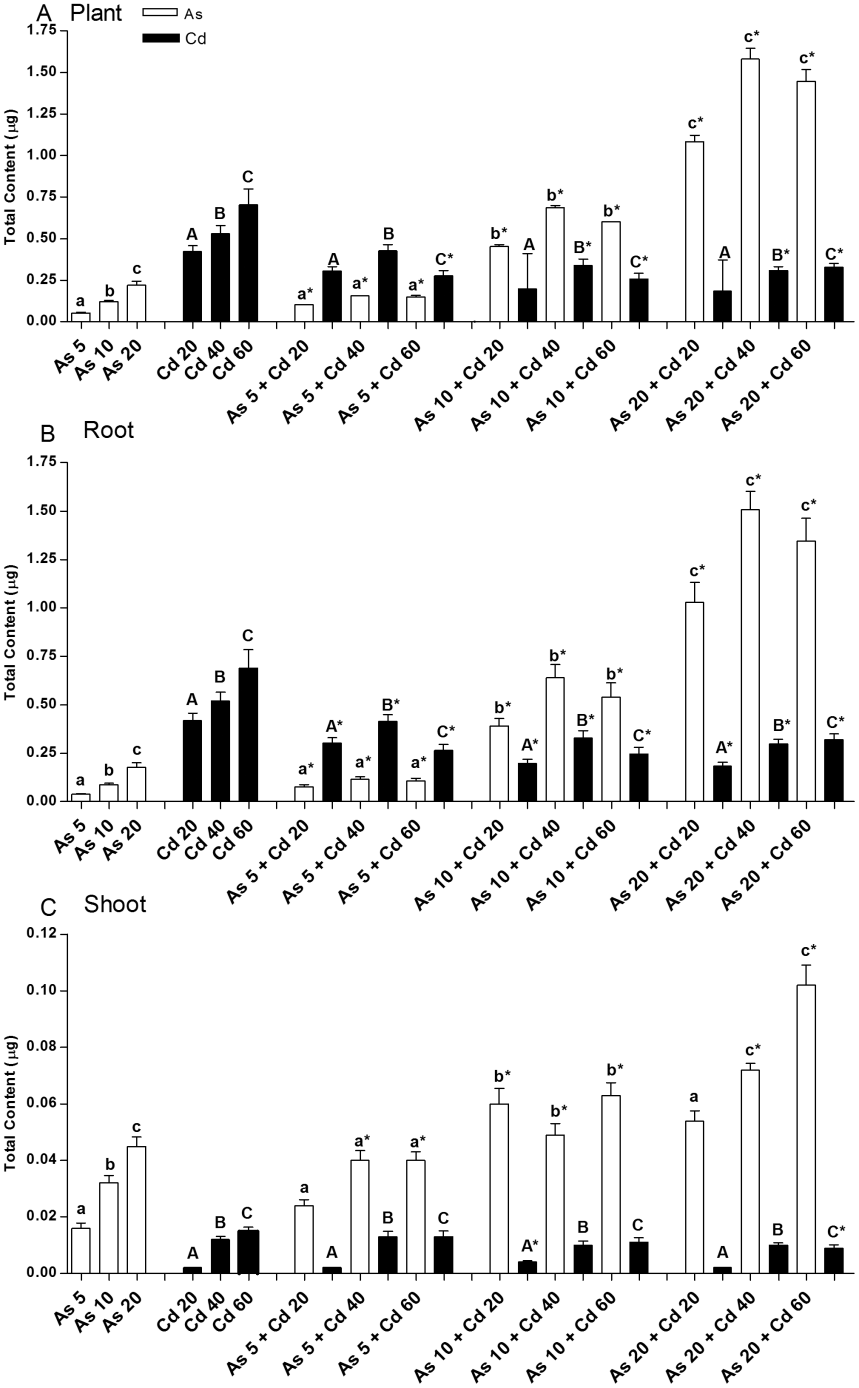
Metal(loid) total content (µg) in *T. repens* plants after exposition. Mean total amount of Cd and As accumulated in plant organs during exposition, was calculated for each treatment by multiplying the metal(loid) concentration, determined by AAS in root and shoot, with the correspondent organ dry weight. Uppercase letters represent significant differences with the correspondent concentration of Cd control (P<0.05); Lowercase letters represent significant differences with the correspondent concentration of As control (P<0.05).

The total amount of Cd and As accumulated in plant organs at the end of the experiment, was calculated by multiplying the element concentration, determined by AAS in root and shoot (Fig.S1), with the correspondent organ DW (Fig.1). The obtained results are reported in Fig.2.

On average, plants grown in soil contaminated with As accumulated an amount of metalloid proportional to the concentration of As added to the soil, which was also related to the amount of bioavailable element (r^2^_bioav-As_ =  0.97, P<0.05). Differently, plants grown in soil contaminated with Cd accumulated a mean amount of metal not proportional to its soil bioavailable concentration; although plants tended to accumulate higher Cd amounts in presence of higher concentrations of Cd in soil, the differences were not statistically significant (Fig.2A). Moreover, with respect to the available amounts of Cd and As, plants accumulated a greater relative amount of As than Cd. Indeed, considering that the available amounts of Cd in each pot containing 2kg of soil were much higher (ranging from about 32 to74 mg) than those of As (ranging from about 0.16 to 1.6 mg ), the relative mean amounts of Cd accumulated per plant (ranging from about 0.4 to 0.7 µg) were proportionally lower than those of As (ranging from 0.05 to 0.2 µg;), suggesting different plant absorbtion mechanisms for the two metal(loid)s.

Similarly, in soils contaminated with both metal(loid)s, the mean total amounts of As accumulated in plants were related to element bioavailability (multiple r^2^ = 0.90, P<0.05) and, since the presence of both metal(loid)s in soil increased As biovailability, its amount, accumulated in plants grown in the presence of both elements, was higher than that measured in the plants grown in presence of As alone. On the contrary, Cd accumulation was not proportional to the bioavailable amount in soil and was lowered by the presence of As (Fig.2A).

Regarding the distribution of Cd and As in plant organs, most of them were accumulated in root (Fig.2B) and the very low amounts translocated to shoot (Fig.2C) were proportional to the amounts accumulated in root (r^2^_Cd_ =  0.51, r^2^_As_ =  0.69, P<0.05).

The same trend of Cd and As accumulation and distribution was also observed analyzing the mean metal(loid) concentration measured in plant organs (Fig.S1). However it can be observed that, due to the different reduction in plant growth, induced by the different metal(loid) treatments, the mean total amount of Cd and As (calculated multiplying metal concentration for DW), did not always reflect the mean concentration of elements in plant organs. For instance, the mean concentration of Cd measured in roots of plants grown in As20+Cd60 soil was statistically higher than that found in root of plants grown in As20+Cd40 soil whereas the mean total amount of Cd was not statistically different between the two treatments, due to the higher growth reduction of plants grown in As20+Cd 60 soil. Thus, in our data elaboration, the total amount of metal(loid)s was calculated to properly correlate the amount of element absorbed by plant with its bioavailable soil quantity, whereas the concentration of elements in plant organs was also taken in to account to better evaluate the observed toxic and genotoxic effects of metal(loid)s.

### Single and joint genotoxic effects of Cd and As

DNA sequence changes were evaluated by means of RAPD analysis, a technique which detects mutations at the primer annealing sites and also within the amplified DNA fragments (*i.e.* deletions or insertions). Twelve single primers were applied for the shoot and root analysis revealing a total of 130 and of 152 reproducible bands, respectively. Of these bands, 3.52% and 4.62% were polymorphic among the shoot and root controls, respectively. These values were considered as a basal polymorphic level among *T. repens* plants (*i.e.* intra-species variability).

**Figure 3:**
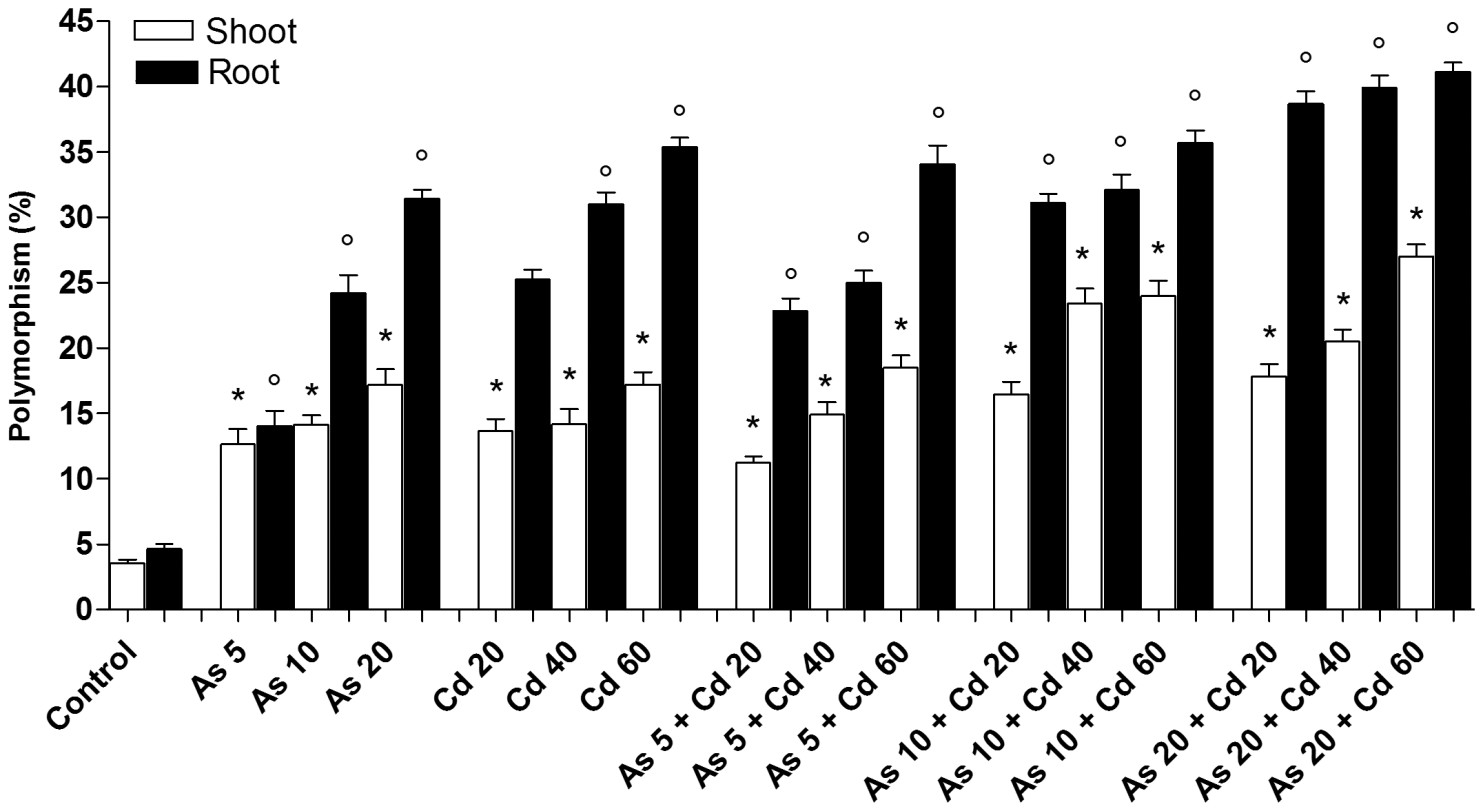
Analysis of the percentage of polymorphism (P%  =  number of polymorphic loci/number of total loci) detected by RAPD in DNA from *T. repens* plants exposed to increasing concentrations of Cd. Root and Shoot mean percentages ± SD for each treatment are reported. The asterisk and circle show statistically significant differences with respect to the control (ANOVA and Dunnet test; P<0.05).

Taking into account all the independent repetitions, DNA sequence damage, induced by Cd and As, was calculated as the percentage of polymorphism (P%) of the treated samples compared to that of the control plants and reported in Fig. 3. All tested As and Cd concentrations (alone or in combination) determined a statistically higher percentage of polymorphisms in the shoots and in the roots compared to the control plants. For both Cd and As, induced plant damage was approximately two-three fold higher in the roots than in the shoots, according to the low amounts of both metal(oid)s translocated to shoot. Moreover, DNA damage was related to the concentration of Cd and As accumulated in shoot and in root. Finally, As was more genotoxic than Cd: 5 µg g^−1^ of As induced a double amount of DNA polymorphisms (14%) than 5 µg g^−1^ of Cd (6%), and 20 µg g^−1^ of As induced a significant higher amount of DNA polymorphism (32%) than 20 µg g^−1^ of Cd (25%).

The interactions between Cd and As, responsible for the joint genotoxic effects observed in Fig. 3, were defined applying the Ince et al. [14] statistical analysis. The results are reported in Table 3. Differently from the interactions responsible for the joint effects on plant development, an antagonistic interaction, leading to a DNA damage reduction, was observed in roots of plants exposed to all the combined concentrations tested. In shoots the interaction was additive except for soils contaminated with the lower Cd concentration (Cd 20) combined with As 5, or As 10, or As 20, which was antagonistic.

**Table 3:** Observed and calculated genotoxic effects at binary test combinations x:y, and single metal concentrations x, y, respectively (predicted interaction types).

	x (µg g^−1^)	y (µg g^−1^)	Observed genotoxicity PP_obs_	Calculated genotoxicityPP_calc_(PP_x_ + PP_y_)	Difference(PP_obs_ -PP_calc_)	Difference standarderror	t Student(df = 4)	Signifi cance(P<0.05)	InteractiveEffect
**SHOOT**	As	Cd							
	5	20	11.3±1.2	26.2±3.7	−15.0	3.9	−.9	S	antagonistic
	5	40	14.9±2.3	26.8±4.1	−11.9	4.7	−2.5	I	addidive
	5	60	18.5±2.3	29.8±3.7	−11.3	4.4	−2.6	I	additive
	10	20	16.5±2.3	27.8±2.9	−11.3	3.7	−3.1	S	antagonistic
	10	40	23.5±2.9	28.3±3.4	−4.8	4.4	−1.1	I	additive
	10	60	24.0±2.9	31.3±2.9	−7.3	4.1	−1.8	I	additive
	20	20	17.9±2.3	30.8±3.7	−13.0	4.4	−3.0	S	antagonistic
	20	40	20.5±2.3	31.3±4.1	−10.9	4.7	−2.3	I	additive
	20	60	27.0±2.3	34.4±3.7	−7.4	4.4	−1.7	I	additive
**ROOT**	5	20	22.8±2.3	39.3±3.4	−16.4	4.1	−4.0	S	antagonistic
	5	40	25.0±2.3	45.0±3.7	−20.0	4.4	−4.6	S	antagonistic
	5	60	34.1±3.5	49.4±3.4	−15.3	4.8	−3.2	S	antagonistic
	10	20	31.1±1.7	49.5±3.9	−18.4	4.2	−4.3	S	antagonistic
	10	40	32.1±2.9	55.2±4.2	−23.1	5.1	−4.6	S	antagonistic
	10	60	35.7±2.3	59.6±3.9	−23.9	4.5	−5.3	S	antagonistic
	20	20	38.7±2.3	56.7±2.4	−18.0	3.4	−5.3	S	antagonistic
	20	40	39.9±2.3	62.4±2.9	−22.5	3.7	−6.1	S	antagonistic
	20	60	41.1±1.7	66.8±2.4	−25.6	3.0	−8.5	S	antagonistic

PP: percentage of polymorphism; S: statistically significant; I: statistically insignificant; df: degrees of freedom.

### RDA analysis

**Figure 4:**
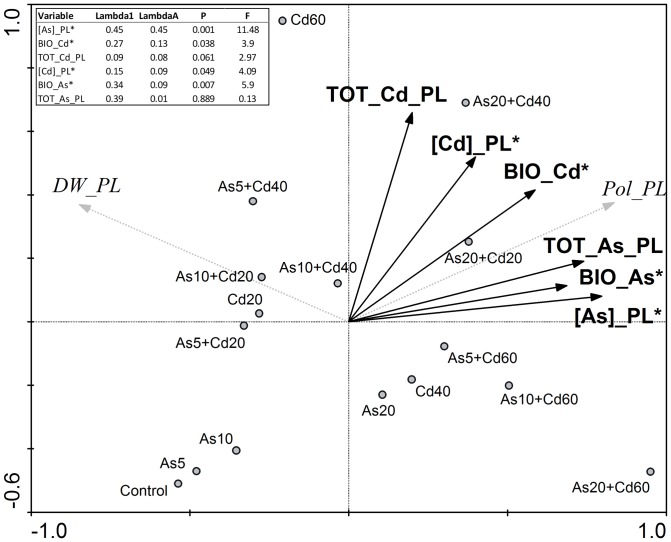
RDA analysis showing the relationship between the metal(loid) effects on plant growth (DW_PL) and DNA sequence (Pol_PL) and the following variables: total content of metal(loid)s in plant (TOT_Cd_PL and TOT_As_PL), concentration of metal(loid)s in plant ([Cd]_PL and [As]_PL). * Statistically different (P<0.05).

In order to better understand the correlation among the soil metal(loid) concentrations, their accumulation in plant organs and their effects on plant growth and DNA sequence, a RDA statistical analysis was carried out. Fig.4 shows that 4 of the 6 variables considered (Cd and As bioavailability, Cd and As concentrations in plant organs) were significant (P<0.05) in determining the toxic and genotoxic effects and that the concentration of As found in plant organs was the most relevant factor (Fig.4 inset).

## Discussion

Cd and As are two of the main environmental contaminants, often occurring simultaneously in polluted sites. Although, their individual toxicity and genotoxicity is well proved, few data are available about their joint effects and in particular no information is available about their joint genotoxic action. In our study we investigated the effect of combined concentrations of Cd and As on the growth and DNA damage of *T. repens*, a sensitive plant to metals, widely used in biomonitoring campaigns. Plants are efficient biondicators to get information on cumulative effects of environmental pollutants. They are used as early warning systems for preventing environment alterations and human diseases. However, given the complexity of the mechanisms causing the final effects, the results obtained through bioindication systems should be better interpreted if the knowledge about the interaction of pollutants had improved.

Individual and joint effects of soil inorganic pollutants on bioindicators depend on different factors. First of all, at soil level, the mobility of chemicals influences the amount of compounds which can be absorbed by test-plant. Nevertheless, the uptake is not only dependent on pollutant bioavailability but it is also dependent on plant uptake mechanisms, which are compound-specific. In addition plant possess detoxification strategies, such as metal exclusion, which influence the final concentration of compounds inside the cells [18], [19]. Finally when two or more compounds are simultaneously present in soil, the toxic final effects depend also on the interaction among pollutants which can occur at all levels.

In our experiment we found that all the concentrations of Cd and As, supplemented alone to the soils did not induce any relevant effect on plant survival and growth, whereas they induced a DNA damage related to the metal(loid) concentration measured in the different plant organs. Moreover, we found that some of the concentrations of Cd and As, supplemented as a mixture to the soils, produced a synergistic effect on plant growth and an antagonistic effect on DNA, suggesting an interaction between the two compounds.

In order to understand the main factors which determined our results, we measured the soil bioavailability of Cd and As and the total amounts, and their concentrations accumulated in the different plant organs.

Concerning soil Cd and As bioavailability, in keeping with literature, Cd was much more bioavailable than As [15]–[18]. The very low availability of As that we measured can be ascribed to the form of As that we used to contaminate the soil (arsenite) along with an alkaline soil pH. In fact, Smith et al. [15] observed that the proportion of arsenite sorbed by soil increased with increasing pH. Specifically they observed that sorption by the soil ranged from approximately 0.80 of added As(III) at low pH, to approximately 0.95 of added As(III) at pH 6 to 7. In addition the low availability of As that we recorded should be related to the DTPA-based method that we used. This method was applied because, according to several studies, it provides the prediction of trace elements uptake by plants from soils. In particular, Karak et al. [20] showed a very high correlation between DTPA-extractable As and the labile pool of As suggesting that the latter is the portion of As most hazardous for human health, due to the possibility of entering the food chain.

Interestingly, for both the metal(loid)s, bioavailability increased with increasing metal concentration in the soils only when the two compounds were used separately, whereas, when they were used as a mixture to contaminate soil, the presence of Cd increased the amount of bioavailable As and, on the contrary, the presence of As reduced Cd bioavailability. The reduction of Cd bioavailability in the presence of As was also observed by Sun and collaborators [17]. This type of result suggests a sort of competition between the two metal(loid)s for binding with soil constituents (clays, Al or Fe or Mn oxides, organic matter, etc.). Generally, both Cd and As retention in soil is due to their primary association to organic matter and amorphous Fe and Mn oxides [9], [20], [21]. It is then likely that in our experiment the interaction between Cd and As, involved these soil constituents. Anyway, given the different characteristic of As and Cd, it is very difficult to shade light on the mechanisms determining the bioavailability changes that we observed when the two compounds were simultaneously present in a soil, therefore further works, beyond the aim of the present study, are needed to clarify the Cd and As sorption-desorption processes.

In our experiment, bioavailability was a very important factor for As accumulation, given the linear correlation found between the total As in plant and the soil As bioavailability.

The result was consistent with previous works [16], [17], [22] showing a significant (p<0.01) correlation between As uptake by plants in various treatments and total soil As. On the contrary, regression analysis indicated that Cd accumulation was not linearly correlated to soil bioavailability. This is also in agreement with previous studies, which showed that the uptake of Cd by plant increases proportionally to increasing soil Cd only up to about 20 mg kg^−1^ above which the trend becomes curvilinear [23]. The different behavior of the two metal(loid)s could be explained by considering their absorption mechanisms. The uptake of Cd from the soil occurs mainly via Ca^2+^, Fe^2+^, Mn^2+^ and Zn^2+^ transporters [24], whereas that of arsenite [As(III)] occurs mainly by diffusion across membrane through members of the NIP (nodulin 26-like intrinsic protein) subfamily of aquaporins [25], [26]. Thus we can assume that in our conditions, the main factor determining As accumulation in *T. repens* was bioavailability, whereas the limiting factor for Cd accumulation was related to the uptake system. Moreover, the possible combination of that fraction of arsenate [As (V)], likely formed in soil from [As (III)], with Cd (Cd^2+^+AsO_4_^3−^ Cd_3_(AsO_4_)_2_) could have decreased the ion exchange on the root surfaces, playing a role in the reduction of Cd uptake, as demonstrated by Liu et al. [4], and explaining the reduction of Cd accumulation we observed in plants grown in presence of both the metal(loid)s.

Interestingly, as shown by RDA analysis, in our study the accumulated total amounts of Cd and As in the plant organs were not statistically significant to explain the observed toxic and genotoxic effects. This because some treatments caused a plant organ reduction, so that the effects were related to the concentration of metal(loid)s measured in plant organs and not to the total absorbed amounts. Specifically As concentration was the most important variable due to both its intrinsic toxicity, that was higher than that of Cd at equal concentration (in agreement with Luan and collaborators [16]), and to its chemical characteristics allowing a plant uptake proportional to soil bioavailability, which was also increased by the presence of Cd in the soil. Moreover, although the concentration of Cd was also important in determining the observed effects, we should consider that, differently from arsenite, which is chemically neutral, a fraction of the total amount of Cd^2+^, accumulated in the different plant organs, was likely stored in cell walls, since the negative charges of the cell wall bind and retain heavy metals [27], [28]. It is one of the several mechanisms evolved by plants to cope with Cd^2+^ toxicity, limiting cellular internalization and its associated toxicity [24], [29].

Concerning the observed toxic effect, a reduction of plant growth was induced by most of the combined concentrations of Cd and As tested. The type of interaction between the two metal(loid)s was additive except for the combinations of the higher Cd concentration (Cd 60) with any As concentration, which were synergistic. Joint plant Cd and As toxicity was previously investigated with contrasting results: Luan and collaborators [16] reported a synergistic effect on soybean plants whilst, Liu et al. [4] and Sun et al. [17] observed an antagonistic effect on wheat and rice biomass production. These opposing results are probably due to the different experimental conditions and to the plant response mechanisms to metal stress, which are species-specific and even development stage and organ specific [7]. *T. repens* is a pollutants-sensitive plant and lack of effective tolerance mechanisms, therefore it is not able to tolerate high metal(loid)s concentrations, whose effect might be exacerbated whenever they act simultaneously. Accordingly, in our experimentation a synergistic effect on plant growth reduction was observed in those plants showing a higher total concentration of metal(loid)s. Likely a consistent inhibition of enzyme activity due to the high Cd and As reactivity to sulfhydryl groups along with oxidative stress and deregulation of homeostasis of essential element or their displacement from protein, primarily due to Cd chemical similarity to Zn, Cu and Fe, led to the inhibition of cellular functions and growth.

In addition, the observed plant growth reduction could be due to an arrest of cell cycle specifically caused by plant in response to high DNA damage induced by high concentrations of metal(loid)s. The temporary inhibition of cell cycle progression and DNA synthesis would provide a longer time for DNA repair and for the production of free radical scavengers. In support of this hypothesis we found an antagonistic genotoxic effect in most of the combined treatments. The antagonism could be also related to the similar genotoxic mechanisms of Cd and As involving the induction of ROS and the inhibition of DNA repair enzymes, which could be reach a maximum in the presence of a defined concentration of metal(loid)s beyond which it does not increase. Anyway, further investigations are needed to clarify the cellular molecular mechanisms involved in the interaction between Cd and As.

In conclusion, our experiment showed that Cd and As can interact at different levels producing additive, synergistic or antagonistic effects. In our experimental condition the presence of Cd increased As soil bioavailability, whereas As presence reduced that of Cd. Nevertheless bioavailability determined the absorption of As but not that of Cd, which was likely limited by its uptake mechanisms. Toxicity and genotoxicity were related to the total concentration of Cd and As in plant organs and As concentration was the most significant variable. Joint effects on plant growth were additive or synergistic, whereas joint genotoxic effects were additive or antagonists. We have supposed that growth reduction was due to both toxic effects of Cd and As and to plant response to high DNA damage, which led to a temporary arrest of cell cycle providing a longer time for DNA repair and for free radical scavenger production. This hypothesis is consistent with the antagonistic genotoxic effect observed in most of the combined treatments. Nevertheless the antagonistic interaction of Cd and As could be also associated to the similar genotoxic mechanisms own of the two metal(loid)s.

## Supporting Information

Figure S1 Metal(loid) concentration (µg g^−1^ dry matter) in white clover plants after exposition. The mean concentration obtained by AAS ± standard deviation for each plant organ and for each soil is shown. Uppercase letters represent significant differences with the correspondent concentration of Cd control (P<0.05); lowercase letters represent significant differences with the correspondent concentration of As control (P<0.05).
(DOCX)

Table S1 Sequences of primers used for RAPD analysis.
(DOCX)
