# Supplementary material for: The Combined Toxic and Genotoxic Effects of Cd and As to Plant Bioindicator Trifolium repens L
Source: PLoS One. 2014 Jun 10;9(6):e99239. doi: 10.1371/journal.pone.0099239 (PMC4051651; doi:10.1371/journal.pone.0099239)
Supplement: Figure S1 — Metal(loid) concentration (µg g−1 dry matter) in white clover plants after exposition. The mean concentration obtained by AAS ± standard deviation for each plant organ and for each soil is shown. Uppercase letters represent significant differences with the correspondent concentration of Cd control (P<0.05); lowercase letters represent significant differences with the correspondent concentration of As control (P<0.05). (DOCX) [file pone.0099239.s001.docx]

**Fig. S1** Metal(loid) concentration (µg g^-1^ dry matter) in white clover plants after exposition. The mean concentration obtained by AAS ± standard deviation for each plant organ and for each soil is shown.

Uppercase letters represent significant differences with the correspondent concentration of Cd control (P < 0.05);

Lowercase letters represent significant differences with the correspondent concentration of As control (P < 0.05).
