# Supplementary figures and images for: The Combined Toxic and Genotoxic Effects of Cd and As to Plant Bioindicator Trifolium repens L
Source: PLoS One. 2014 Jun 10;9(6):e99239. doi: 10.1371/journal.pone.0099239 (PMC4051651; doi:10.1371/journal.pone.0099239)

**Tab. S1** Sequences of primers used for RAPD analysis.


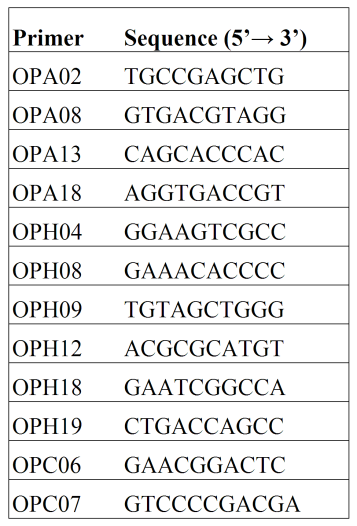

Supplement: Table S1 — Sequences of primers used for RAPD analysis. (DOCX) [file pone.0099239.s002.docx]
